# Emergency Department Utilisation and Related Costs in People With and Without Dementia in Their Last Years of Life

**DOI:** 10.1111/1742-6723.70258

**Published:** 2026-04-13

**Authors:** Namal N. Balasooriya, Tiet‐Hanh Dao‐Tran, Frances Batchelor, Tracy Comans

**Affiliations:** ^1^ Schools of Medicine and Dentistry, Griffith University Gold Coast Australia; ^2^ Centre for Health Services Research, Faculty of Health, Medicine, and Behavioural Sciences, University of Queensland Brisbane Australia; ^3^ Faculty of Health Southern Cross University Gold Coast Australia; ^4^ National Ageing Research Institute Melbourne Australia

**Keywords:** dementia, emergency department utilisation, end‐of‐life care, healthcare costs

## Abstract

**Objectives:**

To compare emergency department (ED) utilisation and related costs between people with and without dementia in their last 3 years of life (including the year of death and two full years prior to the year of death).

**Methods:**

This retrospective study used linked data (2013–2015) from 8389 people with dementia and 7813 people without dementia who died in 2015 in Queensland, Australia. ED utilisation data from the Queensland public hospitals were linked to cost data from the National Hospital Costing Data Collection using the patient's unique identifier. Two‐sample *t*‐test, Chi‐square test and Generalised Estimating Equations regression were used for data analysis.

**Results:**

After adjusting for potential confounders, we show that people with dementia were more likely to have ED presentations (OR = 2.001, *p* < 0.01), short‐stay unit admissions (OR = 1.435, *p* < 0.01) and arrive by ambulance (OR = 2.367, *p* < 0.01) than those without dementia. The average ED cost per episode for people with dementia is AUD 943.24 compared to AUD 912.82 for people without dementia, with a mean difference of AUD 30.43 (*p* < 0.01). ED costs for individuals with dementia were 3% higher, amounting to an estimated AUD 5 million for Australia's health sector in 2023.

**Conclusion:**

People with dementia have higher ED utilisation and costs than those without dementia. The results highlight the need for improved ED care models and targeted resource allocation to accommodate the complex needs of people with dementia.

## 1 Introduction

Dementia is a group of conditions characterised by a gradual decline in brain function beyond normal ageing, potentially affecting memory, cognition, speech, behaviours, mobility and personality [1]. Globally, there were more than 57 million people living with dementia in 2025 [2]. Each year, this number increases by about 10 million [3]. In 2023, Australia had approximately 411,100 people living with dementia [1]. This number is projected to increase to 849,300 by 2058 [1]. Therefore, health services should be set up to provide care to meet the healthcare needs of people with dementia.

Research has shown that people with dementia have more frequent general practitioner (GP) visits than those without dementia [4]. They also have a higher likelihood of being hospitalised even after adjusting for their age, gender and physical comorbidity [5]. Dementia is a predictor of admission to the emergency department (ED) [6, 7]. In EDs, people with dementia often have long waits and experience worrying [8]. Compared to those without dementia, those with dementia have a longer length of stay in the ED [9]. To optimise emergency care for people living with dementia, multiple approaches need to be implemented, including strengthening staff competencies, providing more patient‐centred care interventions and changing environmental systems and policies [10].

In the later stages of dementia, individuals may experience delusions, wandering and hallucinations, contributing to the development of their behavioural crisis [11]. Behavioural crisis presents in the form of aggression and agitation [11], requiring more complex healthcare. People with more severe dementia are more likely to be admitted to the ED [12]. ED staff believe that challenges in caring for people with dementia can be managed with the right resources, but they do not feel prepared to respond effectively [13]. Understanding the use of ED by people with dementia in their later stages and its related cost will provide evidence to adjust resource allocation for their quality of care.

Existing literature has examined ED utilisation among older adults and people with dementia, with systematic reviews identifying a range of clinical, socioeconomic and service‐related factors associated with frequent ED attendance [14, 15]. However, most of this literature has focused on general geriatric populations, where dementia is considered alongside multiple comorbidities or has examined people with dementia without including a direct comparator group. Although there are recent studies focusing on dementia that have primarily investigated predictors of ED attendance near the end of life, they have not focused on ED cost profiles [14]. Based on these latest systematic reviews, we confirmed that there is limited population‐based evidence that compares ED utilisation, episode‐level ED costs between people with and without dementia during the last years of life, particularly in the Australian context.

This study aims to compare ED utilisation and related cost between people with dementia and those without dementia in their last 3 years of life (including the year of death and the previous two full years prior to the year of death). Based on the current literature, the study hypothesised that in their last 3 years of life, those with dementia would have higher ED utilisation [6, 7] and higher related costs than those without dementia.

## 2 Methods

### 2.1 Data

This study includes data from 8389 people living with dementia between January 2, 2013 and December 31, 2015. These people all died in 2015. Their last years of life are defined as 3 years, including the year of death and the two full years preceding it. For comparison, 7813 patients without dementia who visited the ED during this time frame were also extracted. ED visits were defined as any presentation to an ED recorded in the administrative data during the observation period, regardless of presenting complaint or primary diagnosis.

ED presentation data were obtained from the Queensland Emergency Data Collection (EDC), which captures information on all presentations to public hospital EDs across Queensland. The EDC includes demographic information, presentation characteristics, triage category, mode of arrival, ED length of stay and disposition following the ED visit. Costing data for all ED presentations in Queensland public hospitals were extracted from the National Hospital Costing Data Collection (NHCDC). The NHCDC provides episode‐level cost estimates based on the Australian Hospital Patient Costing Standards, including total, direct and overhead costs, derived from locally relevant hospital cost data and does not include out‐of‐pocket costs. Costs were linked to ED utilisation records by the central data linkage team with unique patient identifiers [16]. All costs are reported in Australian dollars (AUD) and reflect the NHCDC price year.

### 2.2 Variables

#### 2.2.1 Outcome Variables

This study has two primary outcomes: ED visits and related costs. In this study, five markers were used for ED visits, including ED visit frequency, ED visits by urgency status, ED arrival modes, ED visit type and ED departure status. The ED visit frequency is the total number of ED visits during the study period. Other ED utilisation variables are nominal, including more than two categories (see Table A1). For the extended analysis, we constructed five binary indicators from the existing ED utilisation variables, indicating whether a patient had (i) an urgent visit (which includes urgent, emergency or resuscitation visits and an unplanned ED visit (Not schedule or referred in advance)), (ii) ED arrival by ambulance (indicating road and air ambulance), (iii) an emergency presentation, (iv) hospital admission following the ED presentation and (v) admission to short stay unit (SSU).

We used three indicators of ED costs: total, direct and overhead episode costs. Direct costs are incurred from the direct departments for the health services event, while overhead costs are from indirect departments that support the delivery of care for the health services event (such as hospital administration, information technology, facilities management and cleaning services). The definition of direct and overhead costs and the costing items are based on the Independent Hospital Pricing Authority Hospital Patient Costing Standards [17]. The total episode cost is the summation of direct and overhead costs.

#### 2.2.2 Variable of Interest

In this study, the variable of interest is dementia status, a binary variable indicating whether the patient had been diagnosed with dementia. Identifying patients' dementia status was not based on the ED department examination; rather, it was determined from ICD‐10‐AM codes (i.e., F00, F01, F02, F03, F05, G30 and G31), which capture a broad range of dementia subtypes, including Alzheimer's disease, vascular dementia and other degenerative dementias. Dementia was therefore treated as a binary exposure in this analysis. Patients with dementia were identified through linkage with the Queensland Hospital Admitted Patient Data Collection (QHAPDC), which records ICD‐10‐AM diagnoses for hospital admissions. Record linkage was performed using patient identifiers and temporal information (year and month), allowing dementia diagnoses recorded in admitted patient data to be linked to ED presentations. Dementia was therefore treated as a binary exposure variable in all analyses.

Both individuals with dementia and those in the comparator group were restricted to a decedent population. For example, both groups consist of individuals who were in their last 3 years of life and had at least one ED presentation during the study period. This ensured that ED utilisation was assessed during comparable end‐of‐life periods for both cohorts, rather than comparing dementia patients with the general ED population.

#### 2.2.3 Control Variables

Age, gender, living area, socio‐economic status (SES), insurance status and cultural background have proven to be associated with ED utilisation [18, 19, 20]. Therefore, we controlled these potential confounding factors in our regression models. We define binary variables for most of our confounding factors, including female, Indigenous status, born in Australia, private health insurance, residence in a major city and low SES. Low SES is defined as a score of Socio‐Economic Indexes for Areas (SEIFA) of three or lower [21]. Additionally, year fixed‐effect was included to control for year‐specific variation.

### 2.3 Statistical Analysis

Stata SE 18.0 was used to perform data analysis. All statistical tests were two‐sided; statistical significance was *p* < 0.05.

#### 2.3.1 Descriptive Analysis

We used descriptive statistics to examine the unadjusted differences in the distribution of outcome variables among people with and without dementia. Initially, Chi‐square tests were applied to ED utilisation variables in their original categorical form (urgency of visit, mode of arrival, type of ED visit and ED departure status). After constructing binary ED utilisation indicators from the original categorical variables, we used two‐sample *t*‐tests to compare outcomes between people with and without dementia.

#### 2.3.2 Model Specification

We specified separate regression models linking each of the five ED utilisation measures and each of the three cost variables using generalised estimating equations (GEE), which account for the correlation within clustered or repeated measures data [22]. Therefore, this approach is suitable considering our data set, which includes multiple ED visits per patient (see Ref. [20]).

We applied GEE Poisson regression with a log link function for cost variables and ED visit frequency and GEE logistic regression with a logit link function [23] to the five binary indicators of ED use: urgent ED visit, unplanned ED visit, arrival by ambulance, hospital admission following ED presentation and short stay unit (SSU) admission. Also, assuming the correlation between any two observations of a patient (within the cluster) is constant, we applied an exchangeable correlation structure across all the models [22, 23]. The general form of our GEE models is specified as follows:

$$g\left(\mu_{\mathrm{ij}}\right)=\beta_{0}+\gamma_{1}{\text{Dementia}}_{\mathrm{ij}}+\sum_{k=1}^{m}\beta_{k}X_{\mathrm{ij}}+\delta_{t}+\varepsilon_{\mathrm{ij}}$$

where *g*(*μ*_*ij*_) is the link function of the outcome variable, *μ*_*ij*_ is the outcome for the *ith* patient in the *jth* visit, *β*_0_ is the intercept, *γ*_1_ is the coefficient of interest, *β*_*k*_ represents the coefficients for the control variables and *δ*_*t*_ and *ε*_*ij*_ represent the year fixed effect and the error term.

Based on our GEE regressions, we estimated the conditional probability of being a dementia patient given ED use, applying Bayes theorem [24]. Bayes' theorem, a robust method for calculating the probability of an event based on prior probabilities of events and new evidence, has been a widely accepted statistical tool [25]. Conditional probabilities of ED use given dementia status and non‐dementia status were obtained from the regression models (Table A4). These estimates were then combined with externally sourced dementia prevalence among individuals aged 65 years and older in Australia in 2023 [1], obtained from the Australian Institute of Health and Welfare, to derive the probability of being a person with dementia given each ED use outcome. Bayes' theorem provides a framework for combining model‐based conditional probabilities with population‐level prior probabilities. No statistical projection or time‐trend modelling was undertaken; alternative dementia prevalence values were examined as scenario analyses to illustrate sensitivity to different assumed prevalence levels. These posterior probabilities were subsequently applied to Australia‐wide ED visit counts for people aged 65 years and over to estimate the number of ED visits and the number of individuals with dementia at the national level.

Unadjusted ED costs in dollar terms were estimated using sample mean costs and compared between patients with and without dementia using two‐sample *t*‐tests. GEE Poisson regression models were used to estimate the adjusted percentage difference in episode costs associated with dementia rather than to generate predicted mean costs. National ED cost estimates attributable to dementia were derived by applying the dementia‐associated percentage increase from the GEE models to the total ED costs of patients with dementia and extrapolating using national ED visit counts and dementia prevalence.

## 3 Results

### 3.1 Sample Characteristics

Table 1 shows the mean values for selected characteristics of ED patients with and without dementia. The results indicate that both groups have a similar proportion of females (48%–49%; mean difference −0.4%, *p* > 0.05) and patients born in Australia (70%; mean difference 0.3%, *p* > 0.05). For other characteristics, the difference between the two groups is statistically significant, for example, compared to the people without dementia, the people with dementia are younger (1.26 years difference, *p* < 0.01), more likely to be of Indigenous ethnicity (0.5% difference, *p* < 0.01), are less likely to live in low socio‐economic areas (1.4% *p* < 0.01) and are in major cities (3.7%, *p* < 0.01).

**TABLE 1:** Mean difference of demographic characteristics between those with and without dementia.

	Mean		Mean difference	Standard error	*t* value	*p*‐Value
	Without dementia	With dementia				
Female	0.484	0.487	−0.004	0.005	−0.750	0.440
Age	85.069	83.809	1.260	0.078	16.150	0.000
Born in Australia	0.699	0.697	0.003	0.005	0.600	0.555
Indigenous	0.014	0.019	−0.005	0.001	−3.950	0.000
Private insurance	0.139	0.153	−0.015	0.004	−4.250	0.000
Low socioeconomic status	0.426	0.413	0.014	0.005	2.900	0.004
Major City	0.599	0.636	−0.037	0.005	−7.950	0.000

*Note:* For binary categorical variables (e.g., sex, Indigenous status, private insurance, socioeconomic status and geographic location), variables were coded as indicator variables (0/1) and the reported means therefore represent proportions. A negative mean difference indicates a lower mean in the non‐dementia group than in the dementia group.

### 3.2 ED Service Utilisation

The chi‐square test results reported in Table A1 indicate that ED utilisation differs significantly between people with and without dementia (*p* < 0.01), with higher utilisation observed among people with dementia, and people with dementia generally have higher utilisation of ED services than people without dementia.

We extend our analysis by considering the most common types of ED use outcomes (such as ED visit frequency, urgent ED visits, ED arrival by ambulance, emergency presentations, hospital admissions and admissions to SSU) to evaluate the unadjusted differences in ED use between people with dementia and those without dementia. The first section of Table 2 shows that patients with dementia have a higher rate of urgent visits (mean difference 1%, *p* < 0.01), using an ambulance (mean difference 11%, *p* < 0.01), emergency presentation (mean difference 2%, *p* < 0.01) and short‐stay admissions (mean difference 4%, *p* < 0.01). Nonetheless, hospitalisation is higher for the people without dementia (mean difference 3%, *p* < 0.01), and the ED visit frequency is also higher among people without dementia (mean frequency is 5.80) than the patients with dementia (mean frequency is 4.71).

**TABLE 2:** Comparison of ED use and costs between people with and without dementia.

	Mean		Mean difference	Standard error	*t* value	*p*‐Value
	Without dementia visits = 20,499	With dementia visits = 24,531				
ED use						
ED visit frequency	5.80	4.71	1.09	0.085	12.95	*p* < 0.01
ED visit Urgent	0.76	0.77	−0.01	0.004	−2.05	*p* < 0.05
ED arrival: ambulance	0.76	0.87	−0.11	0.003	−31.15	*p* < 0.01
Emergency presentation	0.96	0.98	−0.02	0.001	−11	*p* < 0.01
Admitted to hospital	0.55	0.52	0.03	0.005	6.40	*p* < 0.01
Admitted to SSU	0.13	0.17	−0.04	0.004	−12.05	*p* < 0.01
ED cost						
ED cost (DIR)	758.08	781.49	−23.40	4.651	−5.05	*p* < 0.01
ED cost (OH)	154.73	161.77	−7.04	1.073	−6.55	*p* < 0.01
ED total cost	912.81	943.26	−30.45	5.425	−5.60	*p* < 0.01

*Note:* For binary categorical variables (e.g., ED Visit Urgent, ED Arrival: Ambulance, Emergency Presentation, Admitted to Hospital and Admitted to SSU), variables were coded as indicator variables (0/1) and the reported means therefore represent proportions. A negative mean difference indicates a lower mean value in the people without dementia compared to the group with dementia.

Abbreviations: DIR: direct, ED: emergency department, OH: overhead, SSU: short stay unit.

### 3.3 Adjusted Comparisons of ED Visits Between Those With and Without Dementia

We compared ED visits between those with and without dementia by adjusting for other potential confounding factors. Table 3 provides GEE regression estimates to examine the effect of dementia on five dimensions of ED use (e.g., number of ED visits, urgent ED visits, ED arrivals by ambulance, emergency presentations and admissions to SSU). The regression estimates show that people with dementia are significantly associated with higher utilisation of ED services even after controlling for patient characteristics. For example, they are more than twice as likely to arrive at the ED by ambulance (OR 2.334; *p* < 0.01). Similarly, they are 88% more likely to have emergency presentations (OR 1.881; *p* < 0.01), 25% and 41% more likely to be admitted to a SSU (OR = 1.414; *p* < 0.01) compared to people without dementia. On the other hand, patients with dementia are about 14.5% (OR = 0.855, *p* < 0.01) less likely to be hospitalised after ED examination compared to the other patients. However, the difference in ED visit frequency (*β* = −0.207; *p* > 0.1) and urgent ED visits (OR 1.034, *p* > 0.1) is not statistically significant.

**TABLE 3:** GEE regression estimates of ED use on dementia.

	(1)	(2)	(1)	(2)	(1)	(2)	(1)	(2)	(1)	(2)	(1)	(2)
	Freq of ED visit	Freq of ED visit	ED visit urgent	ED visit urgent	ED arrival: ambulance	ED arrival: ambulance	Emergency presentation	Emergency presentation	Admitted to hospital	Admitted to hospital	Admitted to SSU	Admitted to SSU
	*β* (*σ*)	*β* (*σ*)	OR (*σ*)	OR (*σ*)	OR (*σ*)	OR (*σ*)	OR (*σ*)	OR (*σ*)	OR (*σ*)	OR (*σ*)	OR (*σ*)	OR (*σ*)
Dementia	−0.208	−0.207	1.047	1.034	2.207***	2.334***	1.932***	1.881***	0.885***	0.855***	1.377***	1.414***
	(0.139)	(0.114)	(0.046)	(0.041)	(0.111)	(0.108)	(0.300)	(0.249)	(0.026)	(0.025)	(0.047)	(0.047)
Female		−0.099		1.132**		1.169**		1.059		1.059*		1.016
		(0.130)		(0.045)		(0.060)		(0.179)		(0.031)		(0.034)
Age		−0.003		0.998		1.038***		1.013		0.988***		1.018***
		(0.010)		(0.003)		(0.004)		(0.012)		(0.002)		(0.002)
Born Australia		0.105		0.956		1.123*		0.866		0.905***		1.112**
		(0.080)		(0.034)		(0.054)		(0.123)		(0.027)		(0.038)
Indigenous		0.345		0.687*		0.634***		0.914		1.055		0.545**
		(0.293)		(0.121)		(0.084)		(0.204)		(0.183)		(0.112)
Private Insurance		−0.181**		1.135**		1.007		1.135		0.738***		1.093*
		(0.070)		(0.047)		(0.054)		(0.140)		(0.026)		(0.049)
Low SES		0.216*		0.967		0.956		0.791		0.846***		1.258***
		(0.104)		(0.038)		(0.048)		(0.113)		(0.025)		(0.043)
Major City		−0.240		1.670***		1.756***		7.138***		1.099**		1.755***
		(0.124)		(0.067)		(0.089)		(0.823)		(0.035)		(0.068)
Constant	1.758***	1.728	3.132***	2.125**	3.181***	0.079***	25.902***	9.774*	1.207***	1.617**	0.152***	0.016***
	(0.138)	(0.934)	(0.122)	(0.529)	(0.126)	(0.025)	(3.742)	(11.195)	(0.029)	(0.294)	(0.004)	(0.003)
Year FE		Yes		Yes		Yes		Yes		Yes		Yes
*N*	44,030	44,030	44,030	44,030	44,030	44,030	44,030	44,030	44,030	44,030	44,030	44,030

*Note:* In this table, columns 2 through 13 present the coefficients and robust standard errors (in parentheses) for the impact of dementia on different dimensions of ED use. The coefficients of the model for the ED visit frequency are presented as *β* values [*family(poisson) link(log*)], while the coefficients for the other models are presented as OR [*family(binomial) link(logit)]*. Model 1 does not include controls, while Model 2 includes potential controls with year‐fixed effects across all the outcomes to capture the trend. Results are consistent with Pooledaffec Ordinary least square (OLS) regression (Table A2).

Abbreviations: ED: emergency department, FE: fixed effect, GEE: generalised estimating equations, OR: odds ratio, SES: socioeconomic status, SSU: short stay unit.

* *p* < 0.1.

** *p* < 0.05.

*** *p* < 0.01.

Table 4 presents the conditional probability of being a patient with dementia given specific ED use indicators and the estimated number of patients with dementia associated with each indicator. The results show that the chance of being a patient with dementia, given an urgent visit (0.088), arrival by ambulance (0.096), emergency presentation (0.085), hospital admission (0.075) or SSU admission (0.109), is around 8%–11% for the year 2023 in Australia. We predicted ED utilisation of people with dementia at the different levels of dementia prevalence rate. Results showed that ED utilisation rates increase up to 14%–19% if dementia prevalence increases to 15%. Using these conditional probabilities (Table A4) and the total number of ED visits, we estimated that approximately 125,969 and 81,141 people with dementia had urgent visits and hospital admissions, respectively, in 2023.

**TABLE 4:** Population estimates and conditional probabilities.

Measure	ED visit urgent	ED arrival: ambulance	Emergency presentation	Admitted to hospital	Admitted to SSU
$P\left({\mathrm{ED}}_{k}/D\right)$ = Probability of kth ED use given dementia	0.767***	0.878***	0.980***	0.513***	0.174***
$P\left({\mathrm{ED}}_{k}/\mathrm{ND}\right)$ = Probability of kth ED use given non‐dementia	0.725***	0.755***	0.964***	0.551***	0.131***
$P\left(D/{\mathrm{ED}}_{k}\right)=\frac{P\left({\mathrm{ED}}_{k}/D\right)\cdot P\left(D\right)}{P\left({\mathrm{ED}}_{k}\right)}$ = Prob. of being an individual with dementia given a kth ED use					
*P*(*D*) = 0.084^a^	0.088	0.096	0.085	0.079	0.109
*P*(*D*) = 0.900	0.095	0.103	0.091	0.084	0.116
*P*(*D*) = 0.100	0.105	0.114	0.101	0.094	0.129
*P*(*D*) = 0.110	0.116	0.125	0.112	0.103	0.141
*P*(*D*) = 0.120	0.126	0.137	0.122	0.113	0.153
*P*(*D*) = 0.130	0.137	0.148	0.132	0.122	0.166
*P*(*D*) = 0.140	0.147	0.159	0.142	0.132	0.178
*P*(*D*) = 0.150	0.157	0.170	0.152	0.141	0.190
The calculation for the year 2022–2023					
*N* ≥ aged 65^b^	1,431,470	—	—	1,027,105	—
Number of dementia patients^c^	125,969	—	—	81,141	—

*Note: P*(*D*) = 0.07 $P\left({\mathrm{ED}}_{k}\right)$= $P\left({\mathrm{ED}}_{k}/D\right)\cdot P\left(D\right)+P\left({\mathrm{ED}}_{k}/\mathrm{ND}\right)\cdot P\left(\mathrm{ND}\right)$ = Overall probability of a kth ED use. *P*(*D*) and *P*(*ND*) are prior probabilities. *P*(*D*) is the proportion of dementia in the population (prevalence) and *P*(*ND*) = [1 − *P*(*D*)] is the probability of having people without dementia in the population. $P\left({\mathrm{ED}}_{k}/D\right)$ and $P\left({\mathrm{ED}}_{k}/\mathrm{ND}\right)$ are conditional probabilities estimated from GEE models (margin, at dementia 1,0). A detailed table of conditional probabilities is provided in Table A4 of the appendix.

^a^ The dementia prevalence rate among those aged 65 or above in Australia in 2023 [1]. Other *P*(*D*) values are arbitrary.

^b^ The number of ED visits for patients with different ED use outcomes for those aged 65 and older during the year 2022–23 in Australia is sourced from the ED Care data from the Australian Institute of Health and Welfare (AIHW) [26].

^c^ *P*(*D*/*ED*_*k*_) *× N*.

* *p* < 0.1.

** *p* < 0.05.

*** *p* < 0.01.

### 3.4 Costs Analysis

The second section of Table 2 presents the unadjusted differences in ED costs of patients with and without dementia. The results indicate that the average episode ED cost is somewhat higher for patients with dementia. For instance, the average differences are AUD 23 for direct cost, AUD 7 for overhead cost and AUD 30 for total costs per episode. These differences are statistically significant (*p* < 0.001).

Table 5 shows the GEE regression estimates for the three ED cost variables. We show that even after controlling for potential confounders, dementia is significantly associated with increasing costs. Nonetheless, the coefficient for dementia has not changed significantly for either direct or total costs, even when controlling for confounders. The results indicate that having dementia is associated with an approximate 3% increase in episode costs, that is, there is a 3.2% increase in direct ED costs, a 3.4% increase in overhead ED costs and a 3.2% increase in total episode costs for ED visits.

**TABLE 5:** GEE regression estimates of episode cost of ED use on dementia.

	(1)	(2)	(1)	(2)	(1)	(2)
	Direct cost	Direct cost	Overhead cost	Overhead cost	Total cost	Total cost
	*β* (*σ*)	*β* (*σ*)	*β* (*σ*)	*β* (*σ*)	*β* (*σ*)	*β* (*σ*)
Dementia	0.030**	0.032**	0.045**	0.034**	0.033**	0.033**
	(0.011)	(0.011)	(0.014)	(0.012)	(0.011)	(0.010)
Female		0.035***		0.025		0.033**
		(0.010)		(0.013)		(0.010)
Age		−0.001		−0.001		−0.001
		(0.001)		(0.001)		(0.001)
Born Australia		−0.016		−0.042***		−0.021*
		(0.010)		(0.011)		(0.009)
Indigenous		0.205*		0.380*		0.234*
		(0.080)		(0.178)		(0.094)
Private Insurance		0.002		0.010		0.003
		(0.010)		(0.011)		(0.010)
Low SES		−0.069***		−0.120***		−0.078***
		(0.011)		(0.012)		(0.010)
Major City		−0.092***		0.141***		−0.053***
		(0.011)		(0.014)		(0.011)
Constant	6.631***	6.699***	5.042***	5.001***	6.817***	6.868***
	(0.009)	(0.065)	(0.011)	(0.079)	(0.009)	(0.064)
Year FE		Yes		Yes		Yes
*N*	44,030	44,030	44,030	44,030	44,030	44,030

*Note:* In this table, columns 2 through 6 present the coefficients and robust standard errors (in parentheses) for the impact of dementia on costs associated with ED visits. The coefficients are presented as *β* values [*family(Poisson) link(log*)]. Model 1 does not include controls, while Model 2 includes potential controls with year‐fixed effects across all the outcomes to capture the trend. Results are consistent with the Pooled OLS regression results (Table A3).

Abbreviations: ED: emergency department, FE: fixed effect, GEE: generalised estimating equations, SES: socioeconomic status.

* *p* < 0.1.

** *p* < 0.05.

*** *p* < 0.01.

Moreover, Table 6 presents the estimated ED costs related to people with dementia aged 65 and over in Australia for 2022–2023. Our estimation shows that an additional expenditure of approximately AUD 5 million (AUD 4.1 million in direct costs and AUD 0.9 million in overhead costs) was incurred for this population.

**TABLE 6:** ED Cost Estimation Related to Dementia Patients in Australia‐ Year 2022–23.

	(2) Patients aged ≥ 65	Costs			
		(3) Dementia [*P*(*D*) × (2)]	(5) Direct [Mean direct costs × (3)]	(6) Overhead [Mean OH cost × (3)]	(7) Total cost [(5) + (6)]
No. of ED visits	1,989,584	167,125			
Estimated total cost			130,604,845	27,036,647	157,641,492
Additional costs due to Dementia (AUD)^a^			4,179,355	919,246	5,098,601

*Note:* The number of ED visits for individuals aged 65 and above in 2022–2023 is sourced from the ED Care data from the AIHW [26]. *P*(*D*) = (0.084) is the dementia prevalence rate among those aged 65 or above in Australia in 2023 [1]. Mean ED visits (4.7) of dementia patients, mean direct cost (781.48), and OH cost (161.775) are sourced from the sample statistics presented in Table 2.

Abbreviations: ED: emergency department, OH: overhead.

^a^ Additional costs due to dementia = Total ED cost of patients with dementia × *β* (*β* for dementia from Model 2 for each cost category in Table 5).

## 4 Discussion

**FIGURE A1:**
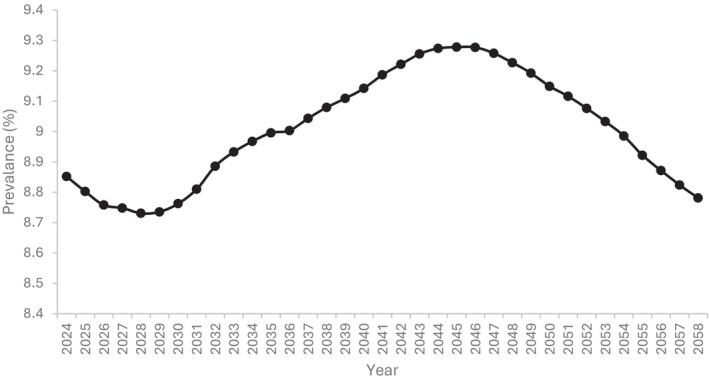
Estimated Dementia prevalence rate, population aged 65 or over in Australia. The calculation is based on the estimated dementia population (Dementia in Australia. (2024). Australian Institute of Health and Welfare, https://www.aihw.gov.au/reports/dementia/dementia‐in‐aus/contents/summary) and the total population projection by the Australian Bureau of Statistics (Population Projections, Australia | Australian Bureau of Statistics. (n.d.). Retrieved June 30, 2024, from https://www.abs.gov.au/statistics/people/population/population‐projections‐australia). We estimated the conditional probabilities of ED use among dementia patients by applying.

This study compared ED use and cost between people with dementia and those without at their last 3 years of life, regardless of the reason for ED presentation. Our findings indicate that people with dementia were more likely to arrive at the ED by ambulance, have emergency presentations and be admitted to the SSU following ED examination compared with individuals without dementia. A detailed cost breakdown showed that dementia is associated with a 3.2% increase in direct ED costs, a 3.4% increase in overhead costs and an overall 3.2% increase in total episode costs. Based on externally sourced dementia population projections [1] and the Australian population forecast [27], we estimate that the prevalence of dementia among individuals aged 65 years and older will increase from approximately 8% to around 10% by 2058 (Figure A1). Under this scenario, our results showed that the probability of using different ED services for an individual with dementia will increase by 10%–14% when the dementia prevalence rate is 10%.

Differences in ED use can be attributed to various health needs. Nevertheless, our findings confirm the existing evidence that individuals with dementia exhibit significantly higher rates of ED utilisation [7, 8, 9, 28]. The lower likelihood and frequency of hospital admissions should be interpreted in the context of differences in ED presentation and admission thresholds at the end of life. Individuals with dementia may present more frequently with behavioural symptoms, functional decline or social crises that do not require inpatient admission, whereas individuals without dementia may present with acute medical conditions more likely to result in hospitalisation [14, 15]. Importantly, this pattern persisted after multivariable adjustment, suggesting it is unlikely to be driven by the definition of the comparator group. However, existing estimates show a higher rate of hospitalisation among individuals with dementia from the ED [28, 29]. Also, in unadjusted analyses, individuals without dementia exhibited a higher frequency of ED visits and higher rates of hospital admission. However, after adjusting for demographic and socioeconomic factors and accounting for repeated visits using generalised estimating equations, there was no statistically significant difference in ED visit frequency between individuals with and without dementia.

Our cost analysis reveals that the ED cost for a patient with dementia is higher than that of a patient without dementia, consistent with previous studies [20, 28, 30]. The higher overhead costs observed among individuals with dementia are likely attributable to the greater complexity of care during ED episodes. Overhead costs reflect indirect hospital services such as administration, infrastructure and support services, which may increase with longer time spent in the ED, greater need for staff coordination, supervision and management of behavioural or cognitive symptoms. These costs are not driven by inpatient length of stay, as hospital admission costs were not included in the analysis.

We also estimated that, for patients with dementia aged 65 and older who visited the ED in 2023, the total adjusted additional cost is approximately $5 million. We suggest that this estimate is robust and reliable, as it is consistent with the observed ED costs of dementia‐related presentations in Australian public hospitals across all age groups in the 2020–2021 financial year, which were estimated at AUD 8.6 million [31].

## 5 Implementation

Our results indicate a significantly higher ED workload associated with dementia patients. Also, we believe that these numbers further increase with the increasing ageing population [32, 33]. Despite the pressure on emergency care [28, 34], increasing ED use of dementia patients correlates with unique challenges of managing patients [35, 36] and is also linked with caregiver depression [37]. Therefore, on the one hand, our results also support proposals highlighting the need for specialised care pathways in the ED, such as developing dementia‐friendly emergency care protocols [10, 38]. Although this study focuses on dementia care in the last years of life, the findings are intended to inform broader ED care pathways for people with dementia who are at higher risk of complex presentations, rather than requiring clinicians to identify end‐of‐life status at presentation. On the other hand, our results have implications for mitigating ED visits by dementia patients, e.g., providing caregivers with necessary resources and training, along with implementing geriatric nursing, high‐intensity telemedicine and community‐based palliative care [19, 39, 40, 41].

Moreover, early diagnosis, proactive management of comorbid conditions and timely access to community‐based support services may help prevent acute health deterioration that often causes emergency care [42, 43]. Interventions that delay disease progression, improve symptom control and enhance continuity of care (such as comprehensive primary care coordination, home‐based support programmes and integrated dementia care models) have the potential to reduce both the frequency and severity of emergency presentations [43, 44]. Collectively, these findings highlight that investments in prevention, early intervention and coordinated care are likely to yield benefits that extend beyond ED settings, supporting more sustainable dementia care as population ageing accelerates.

## 6 Strengths and Limitations

However, given that the study linked recorded data across multiple databases, which helps to minimise the resources (both cost and time) used for data collection rather than primary data collection. Compared with primary data collection, recording data helps reduce recall bias. Linked data encompasses information that may not have been collected in a single database. The data are from the state‐wide population, providing a large dataset with strong statistical power in the data analysis. The dataset contains 16,202 individuals across both samples (8389 individuals with dementia and 7813 individuals without dementia), contributing more than 40,000 ED presentations. The large sample size improves statistical power, enables precise estimation of differences in ED utilisation and costs and supports robust multivariable analyses accounting for repeated presentations.

The data were from people with all types of dementia, helping to minimise selection bias, which often occurs when data come from people with a specific type of dementia. Building upon the bivariate comparison, we further analysed ED utilisation and costs by controlling for potential covariates. We employed GEE regression, which is well‐suited to our data structure (multiple ED visits data). Additionally, as a robustness check, we estimated pooled OLS regression models across all our outcomes (Tables A2 and A3) and demonstrated that the trends are consistent with those from GEE regression estimates.

Additionally, we predicted ED utilisation using conditional probability, calculated based on Bayes' theorem. We also estimated the additional ED utilisation costs attributable to people with dementia by calculating the adjusted cost ratio between individuals with and without dementia using GEE regression.

This study has certain limitations. While we adjusted for sex, socioeconomic status, remoteness and private insurance in multivariable models, we did not conduct stratified or interaction analyses to quantify subgroup‐specific effects; future work should examine heterogeneity to better inform targeted interventions. We used linked administrative data from Queensland hospitals, which does not give details on specific treatments. We also acknowledge that identifying people with dementia relies on ICD‐10‐AM codes rather than direct clinical assessments due to the limitations of the administrative data set. Therefore, we were unable to examine dementia subtypes separately. Additionally, our estimates were not adjusted for detailed clinical factors such as multimorbidity, frailty, behavioural symptoms or functional impairment, as these variables are not available in the dataset and, where present, are likely to lie on the causal pathway between dementia and ED utilisation.

## 7 Conclusion

We provide robust evidence of the increased demand and cost burden associated with dementia in ED settings. Our results show that people with dementia showed greater reliance on ED services, including ambulance use, emergency presentations and SSU, than those without dementia. Dementia was also associated with higher ED costs, reflecting the higher demand on resources during emergency care. As dementia prevalence increases in an ageing population, these patterns of ED utilisation may place increasing pressure on emergency care systems. Developing dementia‐friendly ED care protocols and community‐based support may help manage complex care needs and reduce avoidable emergency presentations.

## Funding

The authors have nothing to report.

## Ethics Statement

This study obtained ethical approval from the University of Queensland's Human Research Ethics Committee (2023/HE001971).

## Consent

No informed consent was required from the participants, as the analysis was performed on de‐identified data.

## Conflicts of Interest

The authors declare no conflicts of interest.

## Data Availability

Research data are not shared.

## Appendix A Tables A1, A2, A3, A4, Figure A1

**TABLE A1:** Type of ED use among people with and without dementia.

Dependent variables	Dementia status			Chi‐square	*p*‐Value
	People without dementia (*n* = 7813)	People with dementia (*n* = 8389)	Total		
ED visit: urgency				193.53	< 0.01
Non‐urgent	847 (4%)	505 (2%)	1352 (3%)		
Semi‐urgent	4111 (20%)	4992 (21%)	9103 (21%)		
Urgent	10,746 (52%)	13,057 (55%)	23,803 (54%)		
Emergency	4148 (20%)	4256 (18%)	8404 (19%)		
Resuscitation	644 (3%)	721 (3%)	1365 (3%)		
Total	20,496 (100%)	23,531 (100%)	44,027 (100%)		
ED arrival mode				992.00	< 0.01
Ambulance (fixed wing)	51 (0.25%%)	67 (0.28%%)	118 (0.27%)		
Ambulance (Helicopter)	40 (0.20%%)	30 (0.13%%)	70 (0.16%)		
Ambulance (Road)	15,505 (76%)	20,501 (87%)	36,006 (82%)		
Other	172 (1%)	133 (1%)	305 (1%)		
Walked‐in/public transport	4731 (23%)	2801 (11%)	7532 (17%)		
Total	20,499 (100%)	23,532 (100%)	44,031 (100%)		
ED visit type				229.36	< 0.01
Dead on arrival	4 (0.02%%)	1 (0.00%)	5 (0.01%)		
Emergency Presentation	19,737 (96%)	23,071 (98.04%)	42,808 (97%)		
Hospital in the home	1 (0.00%)	5 (0.02%)	6 (0.01%)		
Planned return visit	399 (2%)	104 (0.44%)	503 (1%)		
Pre‐arranged admission	358 (2%)	351 (1%)	709 (2%)		
Total	20,499 (100%)	23,532 (100%)	44,031 (100%)		
ED departure status				193.54	< 0.01
Admitted to hospital	11,212 (55%)	12,154 (52%)	23,366 (53%)		
Admitted to hospital in HS	4 (0.02%)	9 (0.04%)	13 (0.03%)		
Admitted to SSU	2702 (13%)	4069 (17%)	6771 (15%)		
Admitted to OW	196 (0.96%)	214 (1%)	410 (0.93%)		
Admitted to the EDS	169 (1%)	236 (1%)	405 (0.92%)		
Dead on arrival (not)	12 (0.06%)	8 (0.03%)	20 (0.05%)		
Did not wait	70 (0.34%)	63 (0.26%)	133 (0.30%)		
Died in the ED	237 (1%)	154 (1%)	391 (0.89%)		
Emergency service completed	4657 (23%)	5383 (23%)	10,040 (23%)		
Left at own risk after	54 (0.26%)	66 (0.28%)	120 (0.27%)		
Returned to hospital	0 (0.00%)	1 (0.00%)	1 (0.00%)		
Transferred to another	1186 (6%%)	1175 (5%)	2361 (5%)		
Total	20,499 (100%)	23,532 (100%)	44,031 (100%)		

*Note:* In this table, columns 2 and 3 present the number of observations for people with and without dementia, respectively; column 4 presents the total number of observations; the Chi‐Sq column presents the Chi‐square statistic; the last column presents the *p*‐value for the Chi‐square test. Row percentages indicate the distribution of each category within the two groups.

Abbreviations: ED: Emergency Department, EDS: Emergency Department/Service, HS: Home Service, OW: Observation Ward, SSU: Short Stay Unit.

**TABLE A2:** Pooled OLS regression estimates of ED use on dementia.

Variables	(1)	(1)	(1)	(1)	Admitted to Hospital	(1)
	Number of visits	Urgent ED visit	ED arrival: ambulance	Emergency presentation		Admitted to short stay unit
Dementia status	−1.091 (0.678)	0.029 (0.006)***	0.121 (0.007)***	0.017 (0.004)***	−0.038 (0.007)***	0.044 (0.004)***
Female	−0.518 (0.654)	0.013 (0.006)**	0.02 (0.007)***	0.001 (0.004)	0.014 (0.007)**	0.002 (0.004)
Age	−0.016 (0.054)	0.001 (0)**	0.006 (0.001)***	0 (0)	−0.003 (0)***	0.002 (0)***
Born Australia	0.525 (0.423)	−0.002 (0.006)	0.016 (0.007)**	−0.003 (0.003)	−0.024 (0.007)***	0.014 (0.004)***
Indigenous	2.912 (2.785)	−0.054 (0.031)*	−0.091 (0.028)***	−0.009 (0.013)	0.013 (0.042)	−0.051 (0.014)***
Private Insurance	−0.829 (0.336)**	0.034 (0.008)***	0.001 (0.007)	0.003 (0.003)	−0.074 (0.008)***	0.011 (0.006)**
	1.148 (0.636)*	0.002 (0.007)	−0.006 (0.007)	−0.006 (0.004)	−0.041 (0.007)***	0.03 (0.004)***
	1.79 (0.514)***	0.005 (0.008)	−0.018 (0.008)**	−0.016 (0.003)***	0.169 (0.008)***	−0.013 (0.006)**
	−1.295 (0.783)*	0.051 (0.007)***	0.083 (0.008)***	0.048 (0.005)***	0.023 (0.008)***	0.069 (0.004)***
Intercept	6.141 (4.827)	0.412 (0.039)***	0.198 (0.047)***	0.919 (0.03)***	0.616 (0.044)***	−0.135 (0.024)***
Observations	44,030	44,026	44,030	44,030	44,030	44,030
*R* ^2^	0.036	0.005	0.056	0.029	0.03	0.02
Year FE	Yes	Yes	Yes	Yes	Yes	Yes

*Note:* Robust standard errors are in parentheses.

* *p* < 0.1.

** *p* < 0.05.

*** *p* < 0.01.

**TABLE A3:** Pooled OLS regression estimates of episode cost of ED use on dementia.

Variables	(1)	(1)	(1)
	ED direct costs	ED overhead costs	Total ED costs
Dementia status	24.951 (8.041)***	5.419 (1.96)***	30.371 (9.531)***
Female	26.892 (8.092)***	4.047 (2.021)**	30.939 (9.642)***
Age	−0.671 (0.551)	−0.095 (0.139)	−0.767 (0.659)
Born Australia	−12.426 (7.452)*	−6.7 (1.722)***	−19.126 (8.705)**
Indigenous	179.415 (77.052)**	65.785 (37.214)*	245.201 (111.045)**
Private Health Insurance	1.253 (8.067)	1.571 (1.711)	2.824 (9.229)
	−53.359 (7.954)***	−18.739 (1.825)***	−72.098 (9.288)***
	2.664 (8.406)	3.444 (1.808)*	6.108 (9.651)
	−71.089 (9.225)***	21.614 (1.96)***	−49.475 (10.657)***
Intercept	814.903 (50.557)***	149.718 (12.511)***	964.621 (60.166)***
Observations	44,030	44,030	44,030
R^2^	0.012	0.029	0.011
Year FE	Yes	Yes	Yes

*Note:* Robust standard errors are in parentheses.

* *p* < 0.1.

** *p* < 0.05.

*** *p* < 0.01.

**TABLE A4:** Conditional probabilities.

		Margin	Delta‐method		*P*>z	[95% conf. interval]	
			Standard error	*z*			
ED Visit Urgent	$P\left({\mathrm{ED}}_{k}/\mathrm{ND}\right)$	0.757	0.007	108.18	0.000	0.743	0.771
	$P\left({\mathrm{ED}}_{k}/D\right)$	0.767	0.003	222.35	0.000	0.760	0.774
ED Arrival: Ambulance	$P\left({\mathrm{ED}}_{k}/\mathrm{ND}\right)$	0.755	0.007	104.41	0.000	0.741	0.769
	$P\left({\mathrm{ED}}_{k}/D\right)$	0.878	0.003	270.00	0.000	0.872	0.885
Emergency Presentation	$P\left({\mathrm{ED}}_{k}/\mathrm{ND}\right)$	0.964	0.004	221.48	0.000	0.955	0.972
	$P\left({\mathrm{ED}}_{k}/D\right)$	0.980	0.001	910.83	0.000	0.978	0.982
Admitted to Hospital	$P\left({\mathrm{ED}}_{k}/\mathrm{ND}\right)$	0.551	0.006	95.6	0.000	0.540	0.562
	$P\left({\mathrm{ED}}_{k}/D\right)$	0.513	0.004	123.79	0.000	0.505	0.521
Admitted to SSU	$P\left({\mathrm{ED}}_{k}/\mathrm{ND}\right)$	0.131	0.003	45.45	0.000	0.125	0.136
	$P\left({\mathrm{ED}}_{k}/D\right)$	0.174	0.003	58.07	0.000	0.168	0.180

*Note:* $P\left({\mathrm{ED}}_{k}/D\right)$ = Probability of *k*th ED use given dementia. $P\left({\mathrm{ED}}_{k}/\mathrm{ND}\right)$ = Probability of *k*th ED use given non‐dementia.
